# Fuelling Neuroblastoma: Genomic Analysis of Ketolytic and Glycolytic Gene Expression in Relation to MYCN Oncogene Amplification, Stage and Prognosis

**DOI:** 10.1002/cnr2.70429

**Published:** 2025-12-19

**Authors:** Joseph W. Molloy, Karl Keogh, Mary‐Kate McLoughlin, Lauren Devitt, David Lee, Eric Downer, David Robert Grimes, Denis Barry

**Affiliations:** ^1^ Discipline of Anatomy, School of Medicine Trinity College Dublin Dublin Ireland; ^2^ Discipline of Physiology, School of Medicine Trinity College Dublin Dublin Ireland; ^3^ Discipline of Public Health and Primary Care, School of Medicine Trinity College Dublin Dublin Ireland

**Keywords:** genomics, glycolysis, ketolysis, metabolism, neuroblastoma

## Abstract

**Background:**

Neuroblastoma (NB) is a childhood cancer of the sympathetic nervous system, and its prognosis is poor. NB cells undergo transcriptional changes to utilise aerobic glycolysis as their primary metabolic pathway, which provides an immediate source of ATP to meet high biosynthetic demands. Alternative metabolic fuel inputs, including ketone bodies which require oxidative phosphorylation, may impact the proliferative capacity of NB.

**Aims:**

In this exploratory study, the expression of glycolytic and ketolytic genes in the context of MYCN oncogene amplification, tumour staging 1–4 and Kaplan–Meier survivability was investigated using the R2: Genomics analysis and visualisation platform (http://r2.amc.nl), database.

**Methods and Results:**

Three NB genomics datasets were assessed in the R2 platform and further analysed in GraphPad Prism to investigate the relationships between glycolytic and ketolytic gene expression and prognosis. Glycolytic gene expression is increased in MYCN amplified, metastatic tumours and is associated with worse event free survival. Ketolytic gene expression is lower in metastatic tumours and is associated with better event free survivability. The glycolytic gene expression profile of NB suggests that elevated levels correlate with a low probability of survival. Ketolytic gene expression patterns suggest a decreased reliance for ketolytic energy, which may be exploited to slow tumourigenic growth.

**Conclusions:**

This study validates glycolytic and ketolytic gene expression profiles in metastatic and MYCN amplified NB tumours and through conditional analysis suggests the potential use of these genes in prognosis prediction. Furthermore, the study highlights the reliability and utility of genomic databases as oncogenomic tools for NB research.

**Abbreviations**
βHB: β‐hydroxybutyrate
AcAc: acetoacetate
ACAT1: acetyl‐CoA transferase
BDH1: βHB dehydrogenase
DNA: deoxyribonucleic acid
ENO1: enolase 1
GAPDH: glyceraldehyde 3‐phosphate dehydrogenase
HK2: hexokinase 2
INSS: International Neuroblastoma Staging System
mRNA: messenger ribonucleic acid
NB: neuroblastoma
OXCT1: 3‐oxoacid‐CoA transferase
OXPHOS: oxidative phosphorylation
PGK1: phosphoglycerate kinase 1
ROS: reactive oxygen species

## 1 Introduction

Neuroblastoma (NB) is the most common extracranial solid tumour in childhood and the most common cancer diagnosed during the first year of life. NB is responsible for a disproportionally high percentage of cancer‐related paediatric deaths and incidence rates can vary from 4.1 to 15.8 per 1 000 000 population [1]. Tumour staging and other prognostic factors are vital in determining severity, treatment, and prognosis [2]. Similar to other malignancies, chemoresistance is an ongoing obstacle and treatment‐related morbidities are core considerations for patient outcomes [3]. Complete regression or spontaneous differentiation may occur in all stages of the disease but primarily in infants with stage 4S, although incidences of regression are rare and difficult to accurately determine [4, 5].

MYCN gene amplification is correlated with advanced stages of disease and is highly predictive of aggressive tumour progression with poor clinical outcome and drug resistance [6]. The MYCN transcription factor promotes a pro‐tumour environment in which NB can proliferate, metastasise and evade immune surveillance, and elicit metabolic reprogramming. 20%–25% of all NB at diagnosis harbour MYCN amplification and this percentage rises in high‐risk and metastatic tumours [7]. Moreover, cancer cells including NB undertake a metabolic shift converting glucose to lactate in the presence of oxygen increasing glycolysis at the expense of oxidative phosphorylation (OXPHOS), a process known as the Warburg effect [8, 9]. This metabolic transition confers a selective advantage by facilitating rapid proliferation, immune evasion, pro‐tumorigenic activity and resistance to altered mitochondrial metabolism, and represents a promising therapeutic target in NB [10, 11].

Carbohydrate restrictive diets, including the ketogenic diet, nutrient starvation, pharmacological interventions and prolonged exercise can lead to an acute increase in both serum ketone body concentration and mitochondrial oxidation of ketone bodies, including β‐hydroxybutyrate (βHB), acetoacetate (AcAc) and acetone [12, 13]. Many cancer cells exhibit a reduced capacity to metabolise ketone bodies due to mitochondrial dysfunction, which can be exploited to reduce blood glucose, elevate mitochondrial reactive oxygen species and slow cancer progression [11, 14, 15, 16].

Cancer genomes can link gene expression with tumour predictors and patient survival [17]. The primary objective of this study was to investigate the expression of glycolytic and ketolytic genes in primary NB tumours. The R2 platform is a user‐friendly oncogenomics analysis and visualisation website hosting gene expression data from tumour datasets. The glycolytic genes, *HK2*, *GAPDH* and *ENO1*, were chosen as they mediate early, mid and final stages of glycolysis respectively, and all three ketolytic genes *BDH1*, *OXCT1* and *ACAT1* were analysed for MYCN amplification, survivability and tumour stage progression in three NB datasets. Both glycolytic and ketolytic genes were associated with MYCN amplification when compared to MYCN non‐amplified tumours. Glycolytic genes were associated with poor survivability and levels were elevated in stage 4 NB when compared to stage 1 NB. ACAT1 and OXCT1 gene expression were associated with better event‐free survivability and levels were reduced in stage 4 when compared to stage 1 NB.

## 2 Methods

### 2.1 NB Dataset Selection

The gene expression of glycolytic enzyme genes *HK2*, *GAPDH* and *ENO1* and the ketolytic enzyme genes *BDH1*, *OXCT1* and *ACAT1* were analysed in the context of MYCN amplification, INSS tumour stage progression and event free survival (Table 1). Three NB datasets were chosen on the basis of dataset size. The R2 Genomics Analysis and Visualisation Platform (https://hgserver1.amc.nl/cgi‐bin/r2/main.cgi) facilitates the storage of tumour and genetic datasets and allows users to perform analysis and visualise data in multiple formats. The data can also be extracted from the platform for statistical analysis. Gene expression was used as a proxy for enzyme activity.

**TABLE 1:** The glycolytic and ketolytic genes used in this study, their corresponding proteins, functions and implications in NB.

Glycolytic genes	Enzyme	Function	Implications in NB
*HK2*	Hexokinase 2	Phosphorylates glucose to glucose‐6‐phosphate [18]	HK2 expression and activity levels are increased in metastatic NB variants influencing malignancy progression [19]
*GAPDH*	Glyceraldehyde 3‐phosphate dehydrogenase	Oxidises glyceraldehyde 3‐phosphate molecules and reduces NAD^+^ to form NADH. Phosphorylates oxidised glyceraldehyde 3‐phosphate to give 1,3‐bisphosphoglycerate [20]	Inhibition of GAPDH in NB induced apoptosis. GAPDH is important in NB cell survival [21, 22]
*ENO1*	Enolase 1	Catalyses the conversion of 2‐phosphoglycerate to phosphoenolpyruvate [23]	ENO1 upregulation can inhibit NB cell proliferation and induce apoptosis [24]

Ketolytic genes	Enzyme	Function	Implications in NB
*BDH1*	βHB‐dehydrogenase	Converts βHB to acetoacetate [25].	Unclear due to BDH1 paradox. Evidence BDH1 can rescue effects of glucose deprivation in specified culture conditions [14]
*OXCT1*	3‐Oxoacid‐CoA transferase	Catalyses the transfer of the CoA moiety from succinyl‐CoA to acetoacetate, producing acetoacetyl‐CoA [26]	Decreased expression in immortalised SK‐N‐AS NB cells potentially leading to decreased viability under ketone supplementation [27]
*ACAT1*	Acetyl‐CoA transferase	Catalyses the condensation of two acetyl‐CoAs to make acetoacetyl‐CoA. Involved in reverse reaction, breaking down acetoacetyl‐CoA into two molecules of acetyl‐CoA [28]	Knockdown of mitochondrial ACAT1 expression using chlorogenic acid leads to NB cell differentiation, displaying antitumour activity [29]

The largest three sample size NB datasets with information related to MYCN status, event free survival, INSS stage and gene expression patterns were selected for analysis (Table S1). The three datasets selected were Asgharzadeh (*n* = 249), Cangelosi (*n* = 786) and Kocak (*n* = 649).

In each dataset, tumour samples were taken at the time of diagnosis before cytotoxic medications were given. The Kocak dataset was composed of 649 primary tumour samples (GEO accession tag GSE45547), and was compiled to investigating the relationship between Hox‐C9 and spontaneous regression in neuroblastoma [30]. The Cangelosi dataset was an amalgamation of four separate datasets and is composed of 786 tumour samples [31]. The four datasets included were RNA‐seq498 (available at the GEO accession tag GSE62564), Agilent709 (ArrayExpress E‐MTAB‐1781), Agilent262 (GEO accession tag GSE120572) and Affymetrix 413 (a combination of six sub‐cohorts). The Asgharzadeh dataset was composed of 249 tumour samples, related to the TARGET analysis of neuroblastoma gene expression. The number of samples in each INSS stage is presented in Table 2.

**TABLE 2:** INSS stage sample sizes (*n* value) of the 5 INSS stages in each dataset.

Dataset	Stage 1	Stage 2	Stage 3	Stage 4	Stage 4S (omitted)	Omitted
Kocak	153	113	92	214	78	6
Cangelosi	143	125	105	320	92	1
Asgharzadeh	30	0	1	216	0	2

High‐risk NB was defined as INSS stage 4 disease and stage 1 as non‐high‐risk disease with or without MYCN amplification. This allowed for the investigation of gene‐expression differences between biologically distinct groups: MYCN amplified high‐risk, MYCN non‐amplified high‐risk, and non‐high‐risk NB. The relationship between pathway activity and event free survival was investigated by generating Kaplan Meier (KM) plots on the R2 Platform. The median gene expression level was used as the cut off modus. The R2 platform tested significance between low and high gene expression groups in KM event free survival probability plots using a log rank test and reported the significance as a Chi‐squared value. Values were considered significant when *p < 0.05. The Asgharzadeh dataset was derived from 168 months of data. In the Kocak dataset, 88 datasets analysed event free survival probability derived from 216 months of data. The Cangelosi datasets were derived from over 240 months of data.

A Cox‐proportional hazards model was next implemented to determine any associations between amplification (amp) status, gene expression and event free survival in the Kocak dataset (Table S2). Cumulative log–log plots of *ACAT1* and *OXCT1* stratified into quartiles showed significant cross‐over at approximately days 100 and 365, suggesting a time‐varying effect in these gene expression profiles (Figure S3). A diagnostic of the uncorrected Cox proportional hazards model confirmed violation of these two gene expression profiles. Accordingly, the hazard model was split into time intervals (*t* ≤ 100, 101 ≤ *t* ≤ 365, *t* > 365) where the proportional hazards assumptions of *ACAT1* and *OXCT1* were shown to hold in each regime (Table S3).

### 2.2 Data Investigation and Transformation

Gene expression data relating to MYCN amplified status and INSS stage was imported into GraphPad Prism (version 9). The R2 Platform generates the expression data with a log2 transformation. The log2 transformed data was subjected to a Kolmogorov Smirnov normality test in GraphPad Prism and visualised by generating histogram plots. It was found that the log2 transformed data often failed the normality test. The R2 database assumes Gaussian distribution of all datapoints and therefore performs a two‐tailed *t*‐test with a parametric (Welch's) correction between two groups. To confirm this, the raw and log2 data were exported and assessed for distribution normality. A large proportion of datapoints for every gene in all datasets did not fit the Gaussian data distribution pattern. Primary human cancer cells display gene expression patterns that are not normally distributed and express a complex, heavy tailed phenotype [32]. One of the most effective ways to account for the difference is by using non‐parametric analytics [32, 33]. The raw data from the platform was exported to GraphPad Prism and its distribution investigated (Figure S1). Data were non‐parametric, allowing use of the Mann–Whitney *U* test to compare medians. The assumptions of the Mann–Whitney *U* test were confirmed using QQ plots.

### 2.3 Gene Expression and MYCN Amplification Status

A Mann–Whitney *U* test was performed in GraphPad Prism. This test can be used to identify a significant difference between the medians of two groups if it is known that both groups have a similar distribution. To achieve these conditions, the untransformed data were used. *p* values were considered significant when *p < 0.05.

### 2.4 Gene Expression and INSS Stage

INSS tumour stage was classified into subgroups: stage 1, stage 2, stage 3 and stage 4. Stage 4S is applicable to children under 1 year of age. Samples belonging to this stage, which are characterised by a favourable prognosis and spontaneous tumour regression, and those that lacked staging, were omitted (Table 2). A Kruskal–Wallis test with a post hoc Bonferroni‐multiple comparisons test was performed in GraphPad prism. To further compare stage 1 and stage 4, a Mann–Whitney *U* test was then performed. *p* values were considered statistically significant when *p < 0.05.

### 2.5 Figure and Graph Generation

Kaplan–Meier curves were generated and extracted from the R2 Platform (Figure S2A–C). All other graphs were generated using GraphPad Prism. To visualise fold changes, gene expression data are presented as a mean log2 transformation of the raw data.

## 3 Results

The expression of glycolytic genes *HK2*, *GAPDH* and *ENO1* were analysed in the context of MYCN amplification, INSS tumour stage progression and overall event free survival using KM plots. To generate KM data, gene expression was categorised into distinct high and low expression groups (Table 3).

**TABLE 3:** Kaplan–Meier event‐free survival probability sample size (*n* value) of each dataset compared in Kaplan–Meier survival probability curves.

Dataset	High expression (*n*)	Low expression (*n*)	Omitted (*n*)
Kocak	238	238	173
Cangelosi	385	384	17
Asgharzadeh	124	123	2

MYCN status was classified into two subgroups: MYCN amplified and MYCN non‐amplified. Samples that lacked MYCN status data were omitted from analysis (Table 4).

**TABLE 4:** The sample size (*n* value) of the MYCN status of each dataset.

Dataset	MYCN amplified (*n*)	MYCN non‐amplified (*n*)	Omitted (*n*)
Kocak	93	550	6
Cangelosi	135	628	4
Asgharzadeh	68	175	6

### 3.1 HK2

**FIGURE 1:**
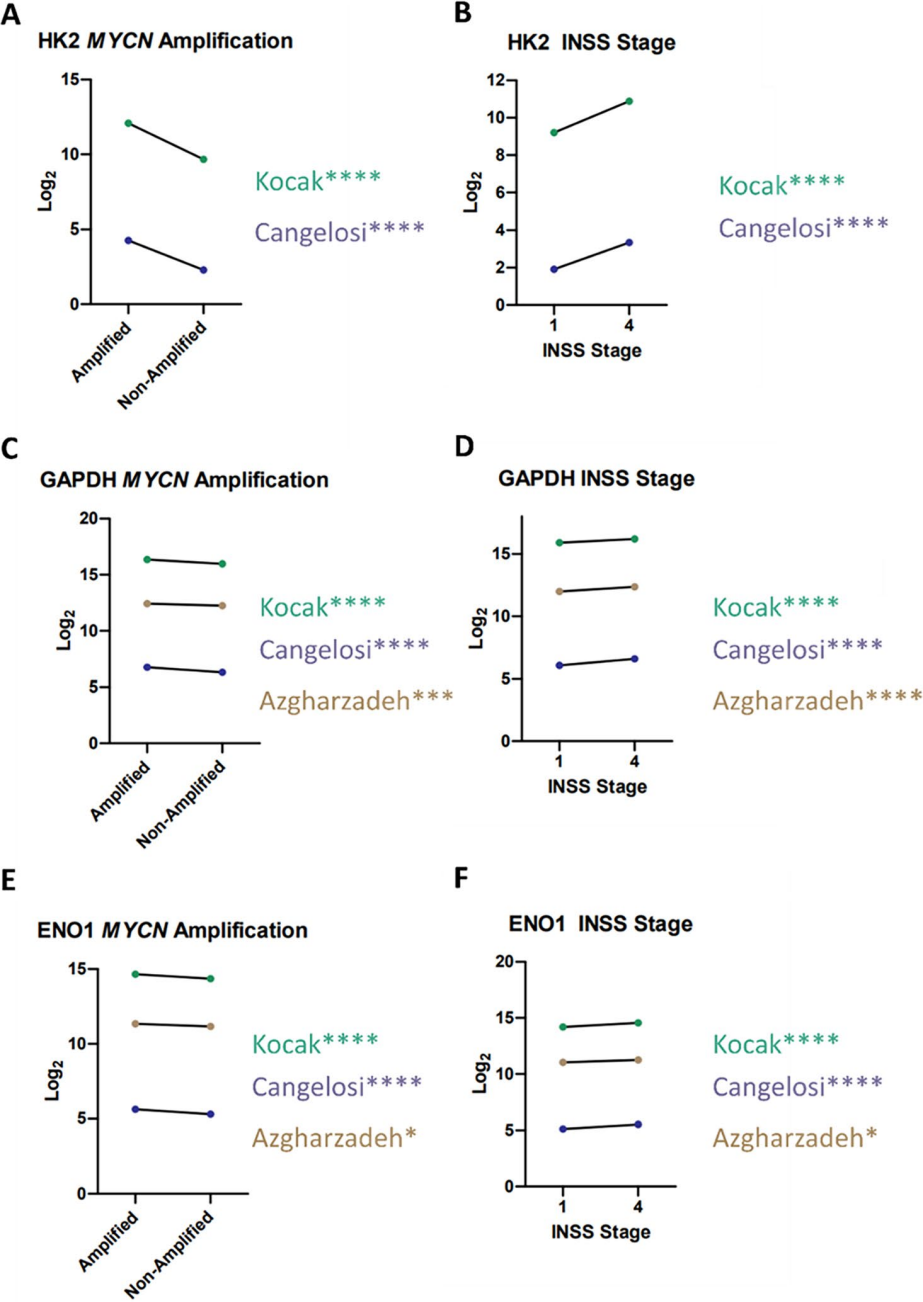
*HK2*, *GAPDH* and *ENO1* expression. (A) *HK2* expression in MYCN and MYCN non‐amplified NB in Kocak and Cangelosi datasets (*p* < 0.0001). (B) *HK2* expression in stage 1 and 4 NB in Kocak and Cangelosi datasets (*p* < 0.0001). (C) *GAPDH* expression in MYCN and MYCN non‐amplified NB in Kocak Cangelosi (*p* < 0.0001) and Azgharzadeh (*p* = 0.0003) datasets. (D) *GAPDH* expression in stage 1 and 4 NB in Kocak, Cangelosi and Azgharzadeh datasets (*p* < 0.0001). (E) *ENO1* expression in MYCN and MYCN non‐amplified NB in Kocak and Cangelosi (*p* < 0.0001) and Azgharzadeh (*p* = 0.0142) datasets. (F) *ENO1* expression in stage 1 and 4 NB in Kocak and Cangelosi (*p* < 0.0001) and Azgharzadeh (*p* = 0.0121) datasets. ****p < 0.0001, ***p < 0.001, *p < 0.05.

*HK2* expression data related to stage, MYCN status and survivability were unavailable for the Asgharzadeh dataset. *HK2* expression is increased in MYCN amplified tumours in Kocak and Cangelosi datasets when compared to MYCN non‐amplified tumours (****p < 0.0001) (Figure 1A). *HK2* expression is increased in stage 4 tumours when compared to stage 1 tumours in Kocak and Cangelosi datasets (****p < 0.0001) (Figure 1B). High *HK2* expression was correlated with poorer event‐free survival in Kocak and Cangelosi datasets (****p < 0.0001) (Table 5).

**TABLE 5:** Correlations of *HK2, GAPDH* and *ENO1* with event free survival probability.

Gene	Dataset	Outcome	*p*	Significant
*HK2*	Kocak	High is worse	*p* = 1.89 × 10^−12^	Yes
	Cangelosi	High is worse	*p* = 1.38 × 10^−22^	Yes
	Asgharzadeh	Not available	Not available	Not available
*GAPDH*	Kocak	High is worse	*p* = 1.30 × 10^−9^	Yes
	Cangelosi	High is worse	*p* = 7.46 × 10^−21^	Yes
	Asgharzadeh	ns	*p* = 0.446	No
*ENO1*	Kocak	High is worse	*p* = 4.43 × 10^−07^	Yes
	Cangelosi	High is worse	*p* = 1.10 × 10^−17^	Yes
	Asgharzadeh	ns	*p* = 0.123	No

*Note: HK2, GAPDH* and *ENO1* from the three datasets demonstrating outcome, *p* value and significance. High is worse: Elevated gene levels correlate with shorter survival in neuroblastoma.

### 3.2 GAPDH

*GAPDH* expression is increased in MYCN amplified tumours in all three datasets when compared to MYCN non‐amplified tumours (****p < 0.0001 Cangelosi, Kocak, ***p < 0.001 Asgharzadeh) (Figure 1C). *GAPDH* expression is increased in stage 4 tumours when compared to stage 1 tumours in all datasets (****p < 0.0001 Cangelosi, Kocak, Asgharzadeh) (Figure 1D). High *GAPDH* expression was correlated with poorer event‐free survival probability in two datasets (****p < 0.0001 Cangelosi, ***p < 0.001 Kocak) (Table 5).

### 3.3 ENO1

*ENO1* expression was increased in MYCN amplified tumours in all datasets when compared to MYCN non‐amplified tumours (****p < 0.0001 Cangelosi, Kocak, *p < 0.05 Asgharzadeh) (Figure 1E). *ENO1* expression is increased in stage 4 tumours when compared to stage 1 tumours in all datasets (****p < 0.0001 Cangelosi, Kocak, *p < 0.05 Asgharzadeh dataset) (Figure 1F). High *ENO1* expression was correlated with poorer event‐free survival probability in two datasets (****p < 0.0001 Cangelosi, **p < 0.01 Kocak) (Table 5).

### 3.4 Expression of Ketolytic Genes in NB

The gene expression of ketolytic enzymes *BDH1*, *OXCT1* and *ACAT1* were analysed in the context of MYCN amplification, INSS tumour stage progression and overall event free survival.

### 3.5 BDH1

**FIGURE 2:**
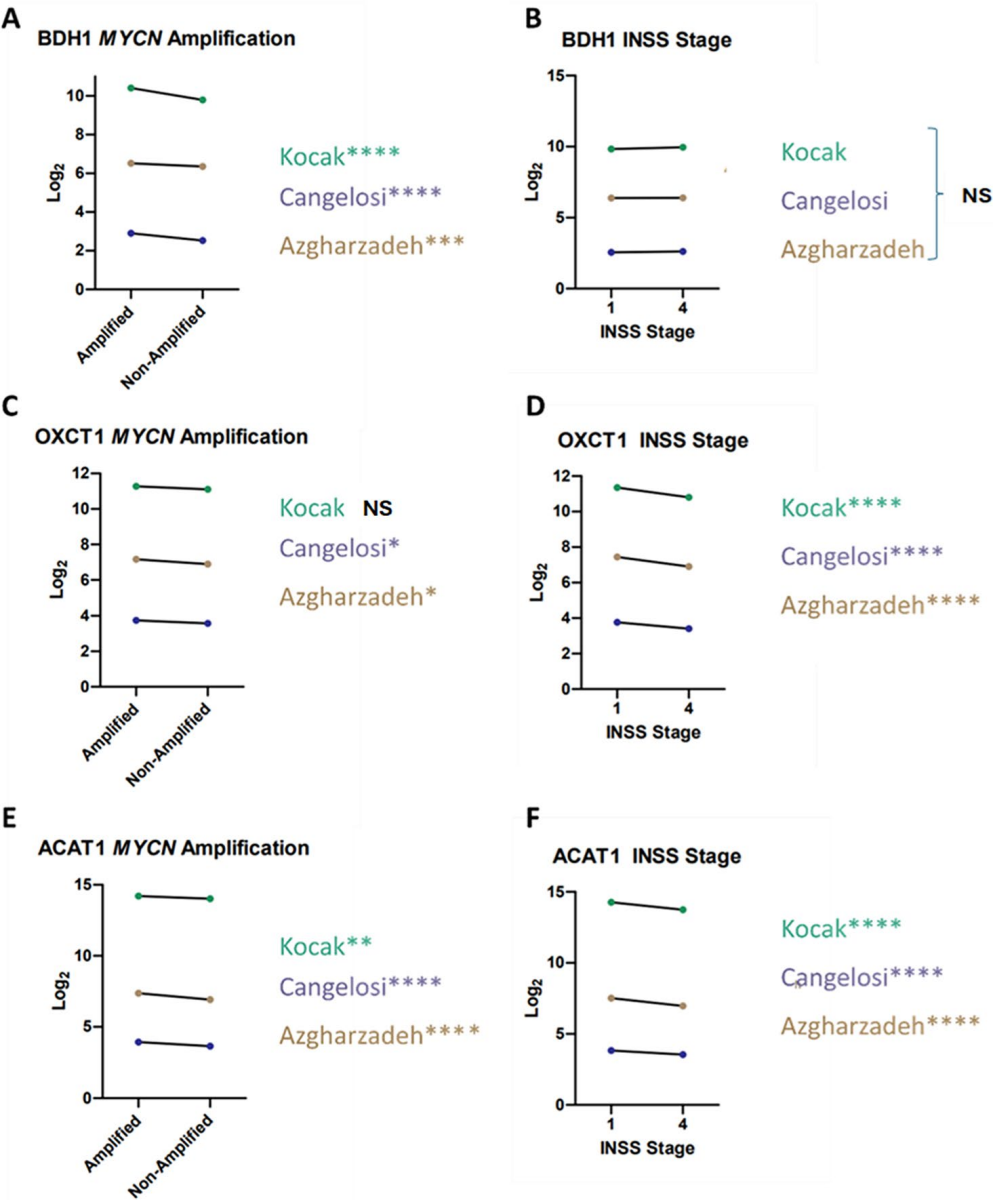
*BDH1*, *OXCT1* and *ACAT1* expression. (A) *BDH1* expression in MYCN and MYCN non‐amplified NB in Kocak, Cangelosi (*p* < 0.0001) and Azgharzadeh (*p* = 0.0004) datasets. (B) *BDH1* expression in stage 1 and 4 NB in Kocak (*p* = 0.0557), Cangelosi (*p* = 0.1451) and Azgharzadeh (*p* = 0.6656) datasets. (C) *OXCT1* expression in MYCN and MYCN non‐amplified NB in Kocak (*p* = 0.1705), Cangelosi (*p* = 0.0182) and Azgharzadeh (*p* = 0.0225) datasets. (D) *OXCT1* expression in stage 1 and 4 NB in Kocak, Cangelosi and Azgharzadeh (*p* < 0.0001) datasets. (E) *ACAT1* expression in MYCN and MYCN non‐amplified NB in Kocak (*p* = 0.0019), Cangelosi and Azgharzadeh (*p* < 0.0001) datasets. (F) *ACAT1* expression in stage 1 and 4 NB in Kocak, Cangelosi and Azgharzadeh (*p* < 0.0001) datasets. ****p < 0.0001, ***p < 0.001, **p < 0.01, *p < 0.05, NS non‐significant.

*BDH1* expression was increased in MYCN amplified tumours when compared to MYCN non‐amplified tumours in all datasets (****p < 0.0001 Cangelosi, Kocak, ***p < 0.001 Asgharzadeh) (Figure 2A). *BDH1* expression was unchanged and not significant in stage 4 tumours when compared to stage 1 NB in all datasets (Figure 2B). High *BDH1* expression was correlated with poorer event‐free survivability in two datasets (****p < 0.0001 Cangelosi, **p < 0.01 Kocak) and unchanged in the Asgharzadeh dataset (Table 6).

**TABLE 6:** Correlations of *BDH1*, *OXCT1* and *ACAT1* with event free survival probability.

Gene	Dataset	Outcome	*p*	Significant
*BDH1*	Kocak	High is worse	*p* = 3.36 × 10^−04^	Yes
	Cangelosi	High is worse	*p* = 7.66 × 10^−7^	Yes
	Asgharzadeh	ns	*p* = 0.141	No
*OXCT1*	Kocak	ns	*p* = 0.334	No
	Cangelosi	ns	*p* = 0.135	No
	Asgharzadeh	ns	*p* = 0.438	No
*ACAT1*	Kocak	Low is worse	*p* = 4.45 × 10^−03^	Yes
	Cangelosi	Low is worse	*p* = 8.75 × 10^−5^	Yes
	Asgharzadeh	Low is worse	*p* = 0.012	Yes

*Note:* Ketolytic genes *BDH1*, *OXCT1* and *ACAT1* from the three datasets demonstrating outcome, *p* value and significance. High is worse: Elevated gene levels correlate with shorter survival in neuroblastoma.

### 3.6 OXCT1

*OXCT1* expression was increased in MYCN amplified tumours when compared to MYCN non‐amplified tumours in Asgharzadeh and Cangelosi datasets (*p < 0.05) (Figure 2C). *OXCT1* expression was decreased in stage 4 NB when compared to stage 1 in all datasets (****p < 0.0001) (Figure 2D). There were no significant differences in event‐free survival between high and low expression in the Kocak, Cangelosi and Asgharzadeh datasets (Table 6).

### 3.7 ACAT1

*ACAT1* expression was increased in MYCN amplified tumours, when compared to MYCN non‐amplified tumours, in all datasets (****p < 0.0001 Asgharzadeh, Cangelosi, **p < 0.01 Kocak) (Figure 2E). *ACAT1* expression was decreased in stage 4 NB when compared to stage 1 NB in all datasets (****p < 0.0001) (Figure 2F). Low *ACAT1* expression was correlated with poorer event‐free survivability in all datasets (****p < 0.0001 Kocak, ****p < 0.0001 Cangelosi, **p < 0.05 Asgharzadeh) (Table 6).

Table 7 shows the hazard ratios of both the time‐split Cox model, including a column on levels of gene expression required to double hazard (for HR > 1) and half hazard (for HR < 1) for genes with significant association with event probability in Kocak datasets. All hazard ratios for gene expression are given per 10 000 units for ease of interpretation. These values were similar to those obtained in the uncorrected Cox model with robust sandwich variance estimator (Table S2). MYCN amplification mutation was a strong predictor of events, with upregulated *BHD1*, *GAPDH* and *EN01* expression associated with event occurrence. *ACAT1* expression had a strong association with a protective effect after 1 year.

**TABLE 7:** Cox‐proportional hazards model to determine associations with MYCN amplification status, gene expression and event free survival in the Kocak data set.

Variable	Hazard ratio (95% CI)	*p*	Level of gene expression required to double/halve hazard
AMP mutation***	2.486 (1.601–3.859)	$p=4.92\times 10^{-5}$	—
*BHD1* expression*	29.64 (2.03–433.64)	p=0.01331	$2.0\pm 1.6\times 10^{3}$
*GAPDH* expression**	1.14 (1.04–1.24)	p=0.00419	$5.0\pm 4.0\times 10^{4}$
*EN01* expression*	1.27 (1.02–1.57)	p=0.03359	$2.9\pm 2.7\times 10^{4}$
*HK2* expression	1.26 (0.83–1.91)	p=0.2859	—
*OXCT1* expression			
*t* ≤ 100	0.40 (0.004–32.94)	p=0.6839	—
100 ≤ *t* ≤ 365	3.91 (0.31–49.66)	p=0.2933	—
*t* > 365	0.09 (0.01–1.41)	p=0.086	—
*ACAT1* expression			
*t* ≤ 100	1.19 (0.76–1.89)	p=0.4299	—
100 ≤ *t* ≤ 365	0.74 (0.50–1.10)	p=0.1369	—
*t* > 365***	0.38 (0.24–0.61)	$p=6.35\times 10^{-5}$	$7.2\pm 3.5\times 10^{3}$

*Note:* Significance: ***p < 0.001, **p < 0.01, *p < 0.05.

## 4 Discussion

Cancer research is placing focus on metabolic inputs by investigating their roles as signalling mediators, drivers of post‐translational modifications and regulators of inflammation and oxidative stress [34, 35]. To further understand the roles of metabolic enzymes in a poorly understood neoplasm with dismal prognostic outcomes, this study showcases the utility of a genomics platform to understand the association between ketolytic and glycolytic gene expression profiles, MYCN amplification, INSS stage progression and event‐free survivability in NB. The three largest, publicly available NB datasets in the R2 platform (Asgharzadeh, Cangelosi and Kocak) were analysed to mitigate biases inherent to single‐dataset analyses. All three ketolytic genes and three early, mid and late‐stage glycolytic genes returned complete genomics data for each parameter tested, with the exception of *HK2* in the Asgharzadeh dataset. The R2 platform was comprehensively searched for *HK2* by inputting the HGNC gene symbol, affymetrix probe cluster ID and its Entrez and Ensembl Gene IDs; however, it appears that the probe cluster for *HK2* was not mapped to any searchable identifier when R2 imported the Asgharzadeh dataset and as such could not be included in this research. Kaplan–Meier and Cox proportional hazards models assessed the prognostic validity of the identified metabolic signatures correlated with MYCN amplification and stage progression. Levels of both ketolytic and glycolytic genes were significantly higher in MYCN amplified tumours when compared to MYCN non‐amplified tumours, with the exception of *OXCT‐1* in the Kocak dataset. Moreover, the glycolytic genes *HK2*, *GAPDH* and *ENO1* showed elevated expression levels in INSS stage 4 tumours when compared to stage 1 tumours, highlighting the increasing energy demands associated with cancer metastasis [36, 37]. The platform also correlated the association of *HK2*, *GAPDH* and *ENO1* expression levels with poorer event‐free survivability in Cangelosi and Kocak datasets. Conversely, *OXCT1* and *ACAT1* expression levels were decreased in INSS stage 4 tumours, when compared to stage 1 in all datasets, indicating the potential reduced reliance of metastatic NB on rate‐limiting ketolytic enzymes under nutrient stress, while *BDH1* levels were unchanged. *BDH1* was associated with poorer survival in Cangelosi and Kocak datasets. *OXCT1* is not associated with survivability in each of the three datasets, while low expression of *ACAT1* was associated with poorer survivability in all datasets and time‐split Cox models suggested protective effects after 1 year where *ACAT1* is expressed. Ketogenic diets are showing promising therapeutic potential in a variety of cancer types [11, 35, 38], while the downregulation of OXPHOS and glycolysis is associated with poorer clinical outcome among some cancers and correlates with a metabolic signature characteristic of the Warburg effect in invasive and metastatic tumours [36, 37]. In NB, the mechanisms regulating metabolic reprogramming are largely unknown and therapeutic interventions can be ineffective in late stage and metastatic cancer. BDH1 and OXCT‐1 have been identified as potential neoplasm biomarkers [39, 40, 41]; however, their mechanistic functions and therapeutic potentials in NB are unknown. ACAT1 has also been found to be involved in tumour progression in a variety of cancer types most likely due to their reliance on cholesterol for membrane biogenesis and various biological processes [42, 43, 44, 45]. Moreover, active ACAT1 acetylates pyruvate dehydrogenase, reducing its activity and potentially decreasing the efficiency of OXPHOS with consequences for the Warburg effect and tumour growth [42], while a recent study described the chlorogenic acid‐induced downregulation of ACAT1 leading to NB differentiation via the metabolic ACAT1‐TPK1‐PDH pathway restricting tumour growth [29], showcasing significant therapeutic promise.

Our data showed MYCN amplification in each of our glycolytic and ketolytic datasets, emphasising its potential significance in NB metabolism [6]. Likewise, multivariate Cox proportional hazard analysis largely confirmed genomic analysis with respect to glycolytic and MYCN amplification expression in stage progression, and that *BDH1*, *GAPDH* and *ENO1* genes were risk factors with respect to event‐free outcome. MYCN is detected in approximately 50% of high‐risk NB cases, although it is unclear when its amplification is initiated and in which cells. Previous genomic datasets demonstrated MYCN amplification as an oncogenic driver in NB, by increasing glycolytic and OXPHOS metabolic capacities to meet increasing energy demands [46]. This analysis supports the identification of metabolically distinct subtypes of NB: MYCN amplified metastatic NB exhibiting elevated glycolytic gene expression, consistent with a Warburg metabolic profile, and non‐high‐risk NB characterised by lower glycolytic activity and conserved or increased ketolytic gene expression, consistent with a less proliferative metabolic phenotype. Taken together, our data support findings that indicate that metabolic reprogramming in NB is not solely determined by MYCN amplification but also by the broader risk context and disease stage [5, 7, 36].

This study utilised genomic analysis to investigate the expression profiles of glycolytic and ketolytic genes in NB, focusing on their associations with MYCN amplification, INSS staging, and event‐free survival. The findings underscore the metabolic reprogramming necessary to fuel NB, particularly the reliance on aerobic glycolysis in high‐risk tumours and suggest potential prognostic utility for these metabolic genes. However, while the study provides valuable insights, several limitations and methodological challenges warrant discussion.

A limitation of this study was the variation in the size of the datasets and differing follow‐up durations across cohorts which may introduce bias or limit prognostic interpretation. The omission of stage 4S tumours, despite their unique biology, may overlook clinically significant metabolic adaptations in cases of spontaneous regression. Moreover, the study does not explicitly address batch effects or normalisation disparities between merged datasets (e.g., Cangelosi's amalgamation of four cohorts), which could confound gene expression comparisons. Furthermore, while glycolytic genes (*HK2*, *GAPDH*, *ENO1*) correlate with poor survival, the analysis cannot establish whether their upregulation drives tumour aggression or is a secondary effect of MYCN‐mediated metabolic reprogramming. Similarly, the protective association of *ACAT1* after 1 year may reflect broader mitochondrial health rather than a direct ketolytic role. For datasets hosted on R2, a manual threshold had to be specified. We chose the default median split as an initial exploratory step, as this subdivides expression into high and low. This is of course arbitrary, and future studies may wish to explore the utility of alternative cut‐off points to enhance data interrogation including scan, average and quartile‐based cut‐off when extracting Kaplan–Meier survivability data. In the full Cox regression analysis, continuous expression level was used in the model, circumventing this limitation. The Cox model violations for *ACAT1* and *OXCT1* necessitated time‐split analyses, suggesting dynamic roles of ketolytic genes over the course of NB progression; however, the biological rationale for these time‐dependent effects is speculative. While this research identifies prognostic markers, it does not validate therapeutic interventions modulating ketolytic or glycolytic genes directly such as the ketogenic diet or glycolytic inhibitors in preclinical models of NB progression. Future proteomic, metabolomic or functional studies (e.g., enzyme activity assays) would strengthen the biological relevance of the extracted datasets and elucidate the metabolic networks driving NB growth.

This study highlights the utility of genomic platforms like R2 in identifying metabolic signatures linked to NB growth, aggressiveness and prognosis. While the glycolytic and ketolytic gene profiles offer promising prognostic insights, their clinical translation requires further mechanistic and therapeutic validation. Addressing these limitations, including dataset heterogeneity and lack of functional data, is critical for advancing metabolic targeting strategies in high‐risk NB.

## Author Contributions

**Joseph W. Molloy:** writing and analysis. **Karl Keogh:** writing, analysis and methodology. **Mary‐Kate McLoughlin:** writing, analysis and methodology. **Lauren Devitt:** writing, analysis and methodology. **David Lee:** analysis and methodology. **Eric Downer:** editing, analysis and methodology. **David Robert Grimes:** writing, analysis and methodology. **Denis Barry:** conceptualization, writing, review and editing, analysis supervision.

## Funding

The authors have nothing to report.

## Ethics Statement

The authors have nothing to report.

## Consent

The authors have nothing to report.

## Conflicts of Interest

The authors declare no conflicts of interest.

## Supporting information


**Data S1:** cnr270429‐sup‐0001‐Supinfo.docx.

## Data Availability

The data that support the findings of this study are available in R2 Genomics Analysis and Visualisation Platform at https://hgserver1.amc.nl/cgi‐bin/r2/main.cgi?open_page=login. These data were derived from the following resources available in the public domain: https://hgserver1.amc.nl/cgi‐bin/r2/main.cgi?open_page=login, https://hgserver1.amc.nl/cgi‐bin/r2/main.cgi?open_page=login.
