# Supplementary material for: Fuelling Neuroblastoma: Genomic Analysis of Ketolytic and Glycolytic Gene Expression in Relation to MYCN Oncogene Amplification, Stage and Prognosis
Source: Cancer Rep (Hoboken). 2025 Dec 19;8(12):e70429. doi: 10.1002/cnr2.70429 (PMC12717149; doi:10.1002/cnr2.70429)
Supplement: Supplementary file 1 — Data S1: cnr270429‐sup‐0001‐Supinfo.docx. [file CNR2-8-e70429-s001.docx]

**Supplementary tables and figures**

**Table 1.** Datasets analyzed, original DOI and R2 platform internal identifier.

| **Gene** | **Method used** | **Original Article DOI** | **R2 internal identifier** |
| --- | --- | --- | --- |
| Asgharzadeh | data was derived from level 3 BER transcript data set | <https://dx.doi.org/10.1186%2F1755-8794-4-35> | ps_avgpres_targetnrbl249_huex10t |
| Cangelosi | Different platforms integrated | <https://dx.doi.org/10.1038%2Fs41467-021-21247-8> | ps_avgpres_dgc2102a786_dgc2102 |
| Kocak | 44K oligonucleotide microarrays | <https://dx.doi.org/10.1038%2Fcddis.2013.84> | ps_avgpres_gse45547geo649_ag44kcwolf |

**Figure. 1.** Distribution of gene expression data and transformations performed.

Gene expression data was visualized in both the log2 transformed and raw forms. As seen in the example of ALDOA in SEQC ag44kcwolf datasets (dataset not analyzed in present glycolytic/ketolytic analysis), the data showed significant deviation from the expected Gaussian distribution.

**Figure 2A**. Kaplan-Meier Survivability Curves. Event-free survival probability for genes: *HK2, GAPDH, ENO1, BDH1, OXCT1,* and *ACAT1* in the Kocak dataset. N = number of tumours.


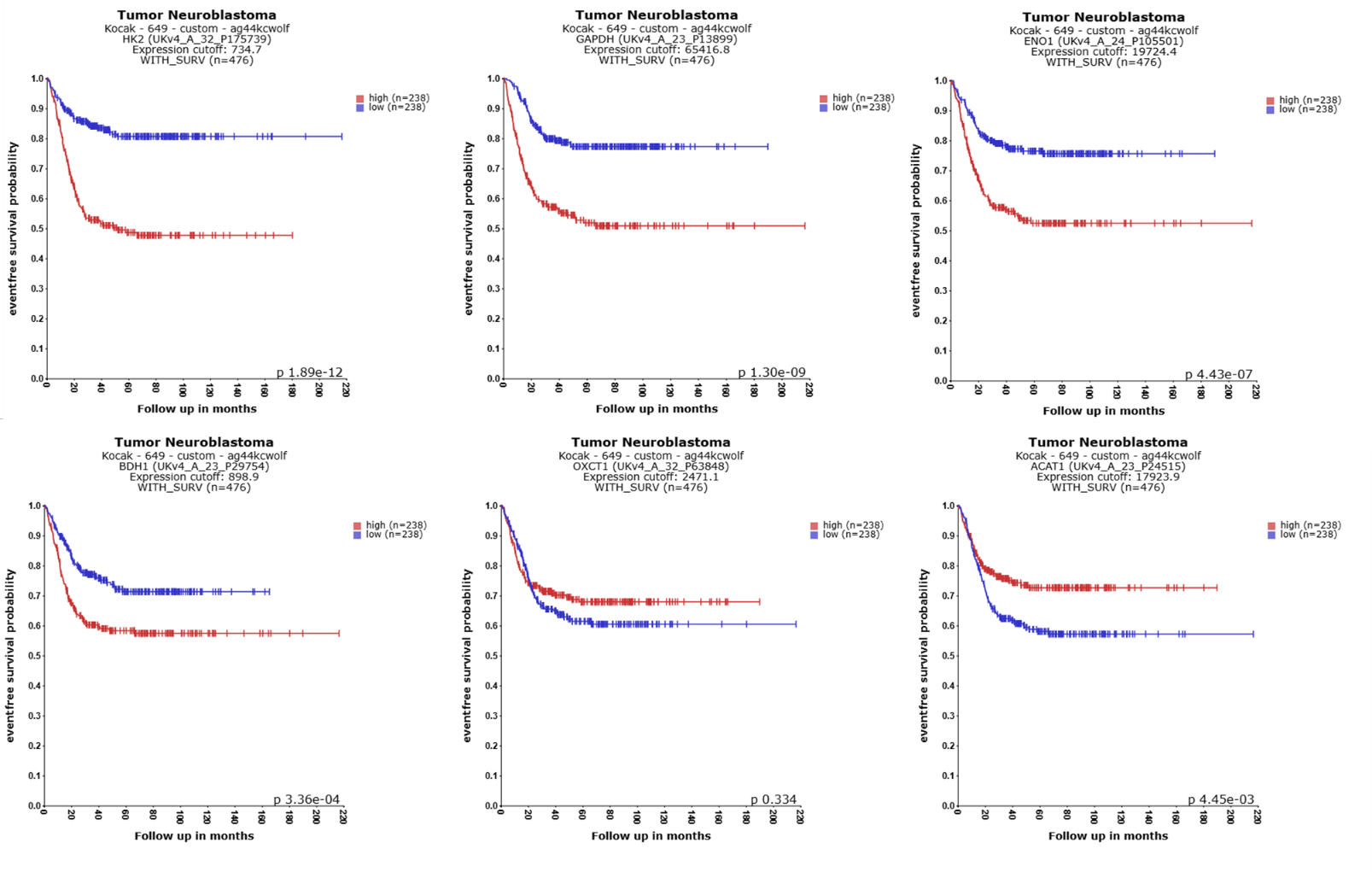


**Figure 2B.** Kaplan-Meier Survivability Curves. Event-free survival probability for genes: *HK2, GAPDH, ENO1, BDH1, OXCT1,* and *ACAT1* in the Cangelosi dataset. N = number of tumours


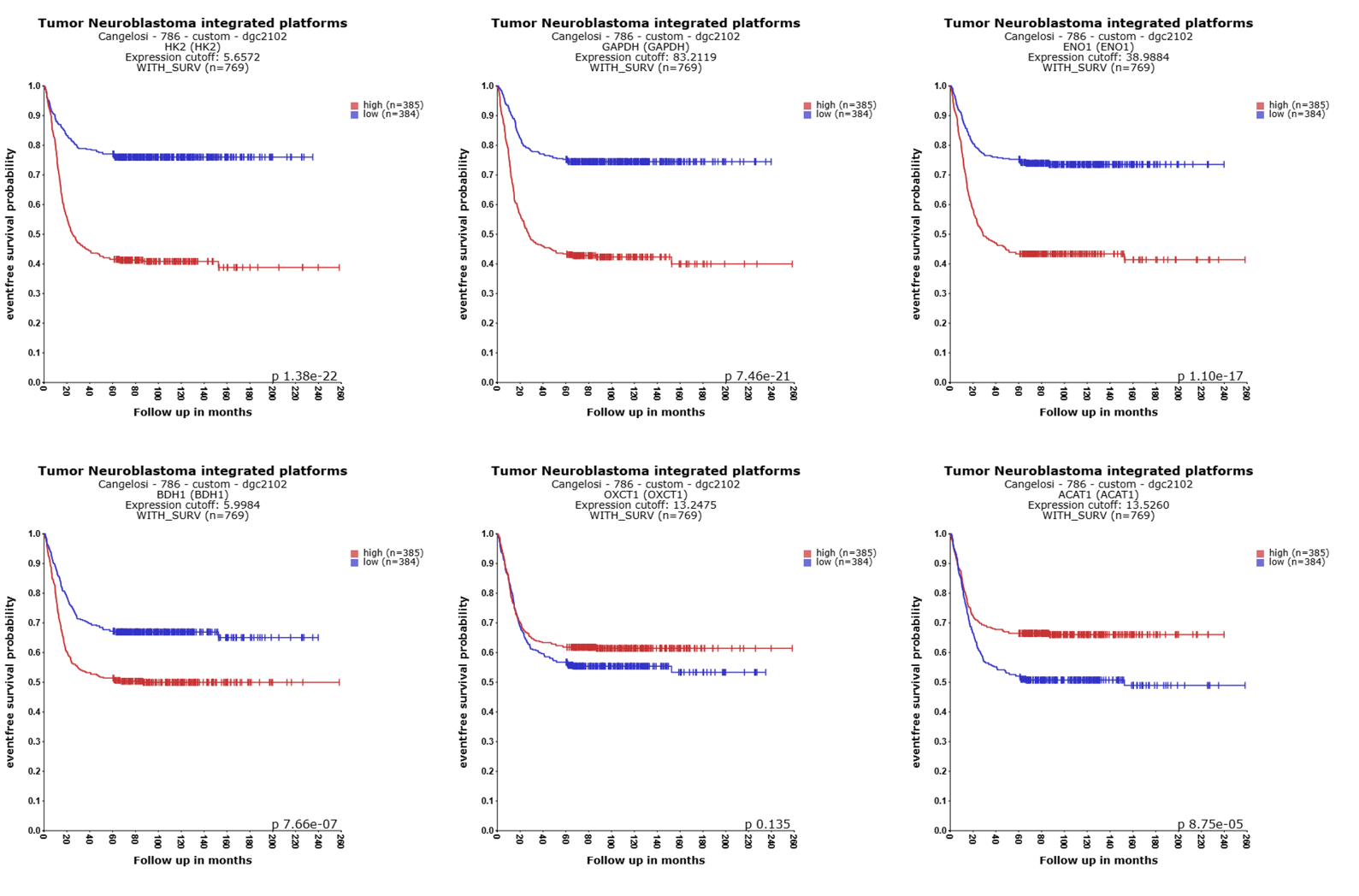


**Figure 2C**. Kaplan-Meier Survivability Curves. Event-free survival probability for genes*: GAPDH, ENO1, BDH1, OXCT1,* and *ACAT1* in the Asgharzadeh dataset (*HK2* unavailable). N = number of tumours.


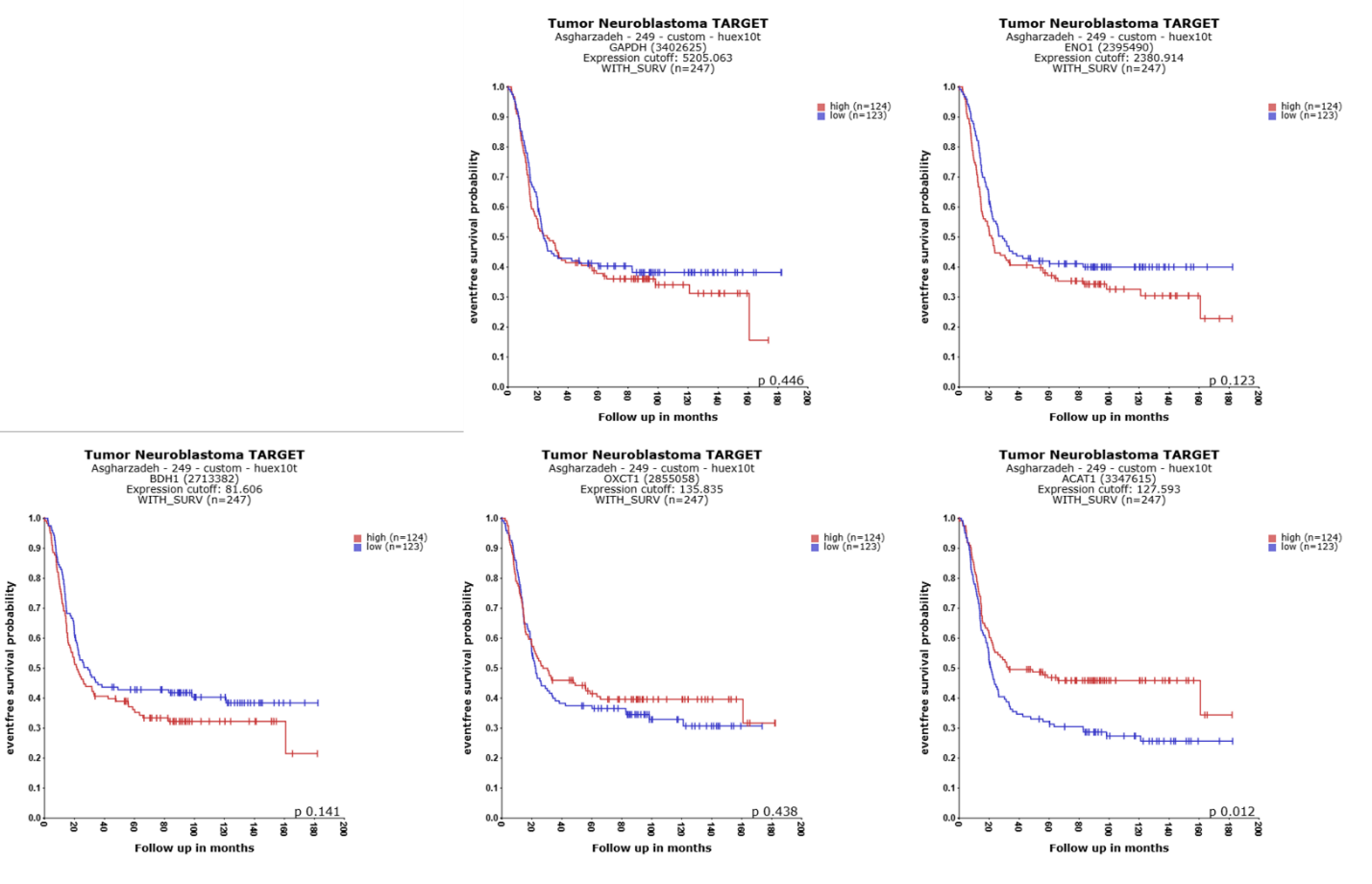


**DATA UNAVAILABLE**

**Table 2**. Cox-proportional hazards model to determine associations with amp status, gene expression, and event free survival in the Kocak dataset. Significance: ***P < 0.001, ** P < 0.01, * P < 0.05

| Variable | Hazard Ratio (95% CI) | P-value |
| --- | --- | --- |
| AMP mutation*** | $2.14 (1.404-3.262)$ | $P=4.01 \times{10}^{-4}$ |
| *BHD1* expression* | $115.93$  $(10.96-1226.69)$ | $P=7.85 \times{10}^{-5}$ |
| *GAPDH* expression** | $1.16$  $(1.06-1.26)$ | $P=7.90 \times{10}^{-4}$ |
| *EN01* expression | $1.22$  $(0.99-1.50)$ | $P=0.5549$ |
| *HK2* expression | $1.31$  $(0.86-2.00)$ | $P=0.2080$ |
| *OXCT1* expression | $0.29$  $(0.05-1.50)$ | $P=1.41 \times{10}^{-4}$ |
| *ACAT1* expression* | $0.61$  $(0.48-0.80)$ | $P=0.1380$ |

*Note: All Hazard Ratios for gene expression are given per 10,000 units for ease of interpretation*

**Figure 3**. Cumulative Log-Log Plot for ACAT1 and OXCT1 showing Cox-PR violations (crossing of quartiles) and approximate time intervals where Cox-PR assumptions hold.


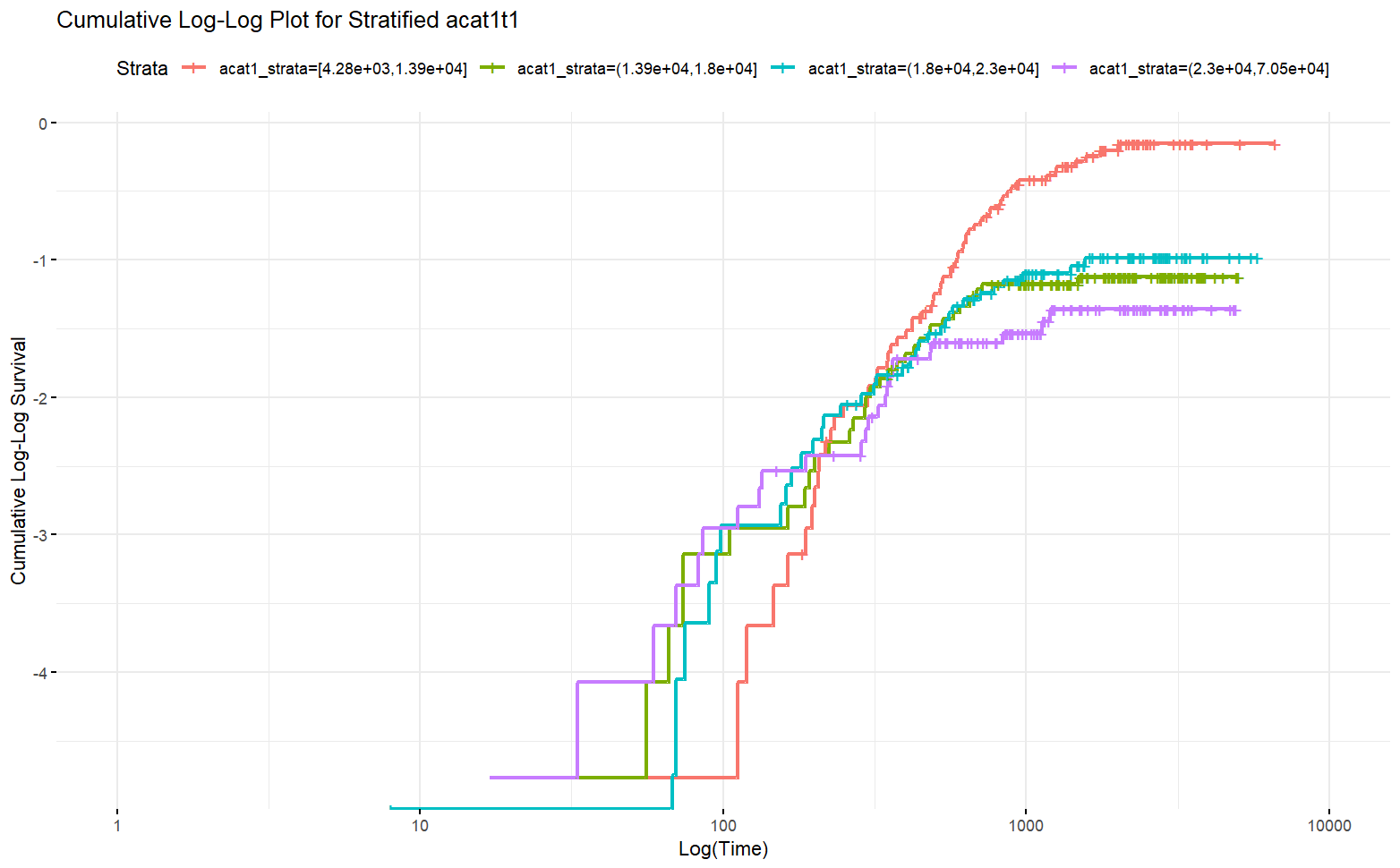

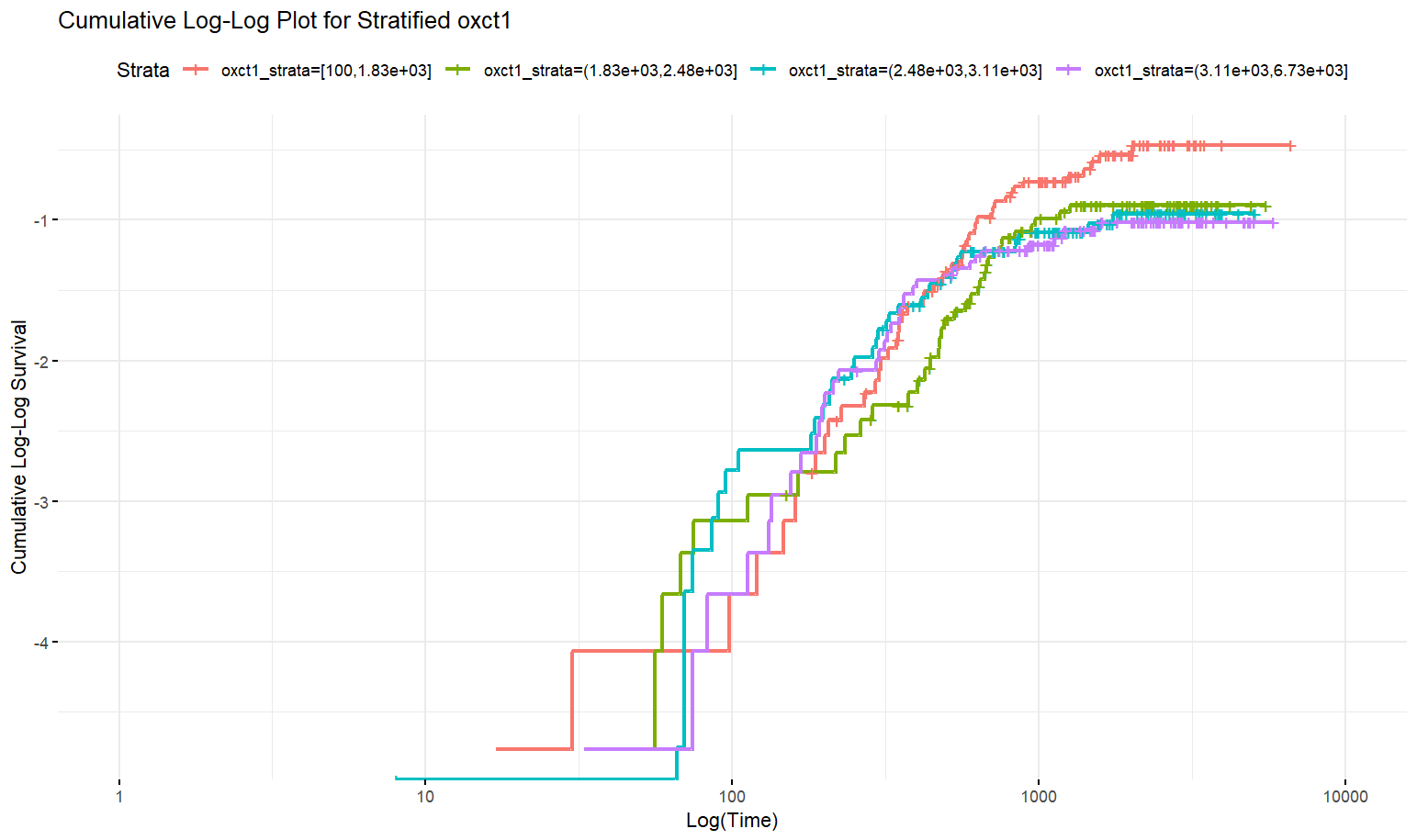


**Table 3**. Cox-proportional hazards model comparison with and without interval adjustment (with violation of Cox-PR assumptions). Significance: ***P < 0.001, ** P < 0.01, * P < 0.05

| Variable | Time Stratified Model Hazard Ratio (95% CI) | Unadjusted Model  Hazard Ratio (95% CI) |
| --- | --- | --- |
| AMP mutation | $2.486 (1.601-3.859)$*** | $2.1406 \left( 1.401-3.2623 \right)***$ |
| *BHD1* expression | $1.000339*$  $(1.00007-1.00061)$ | $1.000474***$  $(1.000239-1.000712)$ |
| *GAPDH* expression | $1.0000128**$  $(1.000004-1.000022)$ | $1.0000146***$  $(1.000006-1.000023)$ |
| *EN01* expression | $1.0000236*$  $(1.000002-1.000045)$ | $1.0000201$  $(0.999999-1.0000406)$ |
| *HK2* expression | $1.0000229$  $(0.99998-1.00006)$ | $1.0000207$  $(0.99998-1.00007)$ |
| *OXCT1* expression |  |  |
| $\boldsymbol{t\leq100}$ | $0.9999094$  $(0.99947-1.00035)$ | $0.999874$  $(0.99970-1.00004)$ |
| $\boldsymbol{100\leq t\leq365}$ | $1.0002363$  $(0.99988-1.00039)$ | $0.999874$  $(0.99970-1.00004)$ |
| $\boldsymbol{t>365}$ | $0.9997593$  $(0.99948-1.000034)$ | $0.999874$  $(0.99970-1.00004)$ |
| *ACAT1* expression |  |  |
| $\boldsymbol{t\leq100}$ | $1.0000182$  $(0.99997-1.000063)$ | $0.999502***$  $(0.999260-0.999703)$ |
| $\boldsymbol{100\leq t\leq365}$ | $0.9999703$  $(0.999931-1.000009)$ | $0.999502***$  $(0.999260-0.999703)$ |
| $\boldsymbol{t>365}$ | $0.99990402***$  $(0.999857-0.999951)$ | $0.999502***$  $(0.999260-0.999703)$ |

**Footnotes**

HK2 expression data related to stage, MYCN status and survivability were unavailable in the Asgharzadeh dataset.

High is worse: elevated gene levels correlate with shorter survival in neuroblastoma.
